# Adaptive SNN for Anthropomorphic Finger Control

**DOI:** 10.3390/s21082730

**Published:** 2021-04-13

**Authors:** Mircea Hulea, George Iulian Uleru, Constantin Florin Caruntu

**Affiliations:** Faculty of Automatic Control and Computer Engineering, Gheorghe Asachi Technical University of Iasi, 700050 Iasi, Romania; george-iulian.uleru@academic.tuiasi.ro (G.I.U.); caruntuc@ac.tuiasi.ro (C.F.C.)

**Keywords:** spiking neural networks, neuromorphic hardware, Hebbian learning, anthropomorphic finger

## Abstract

Anthropomorphic hands that mimic the smoothness of human hand motions should be controlled by artificial units of high biological plausibility. Adaptability is among the characteristics of such control units, which provides the anthropomorphic hand with the ability to learn motions. This paper presents a simple structure of an adaptive spiking neural network implemented in analogue hardware that can be trained using Hebbian learning mechanisms to rotate the metacarpophalangeal joint of a robotic finger towards targeted angle intervals. Being bioinspired, the spiking neural network drives actuators made of shape memory alloy and receives feedback from neuromorphic sensors that convert the joint rotation angle and compression force into the spiking frequency. The adaptive SNN activates independent neural paths that correspond to angle intervals and learns in which of these intervals the rotation the finger rotation is stopped by an external force. Learning occurs when angle-specific neural paths are stimulated concurrently with the supraliminar stimulus that activates all the neurons that inhibit the SNN output stopping the finger. The results showed that after learning, the finger stopped in the angle interval in which the angle-specific neural path was active, without the activation of the supraliminar stimulus. The proposed concept can be used to implement control units for anthropomorphic robots that are able to learn motions unsupervised, based on principles of high biological plausibility.

## 1. Introduction

In the biological world, information is processed using impulses or spikes that provide living creatures with the ability to be aware of the surrounding environment and to act accordingly. For most of the aspects of life, they still outperform conventional, state-of-the-art, robots in terms of speed and energy efficiency [1]. Modelling the motor skills of the human hand and fingers represents a challenging task in robotics, due to the smoothness and diversity of natural motions. The design of control devices for such robotic hands should be based on modelling the behaviour of motor neural areas (MNA) and their bidirectional communication with the muscles. The natural MNA stimulates the muscles through efferent neural pathways that include the motor cortex and the central pattern generators. In the opposite direction, the MNA receives information from spindles about the muscle stretch during relaxation [2] through afferent pathways, and from the Golgi tendon organs during contraction [3]. Considering that the frequency generated by the spindles increases with the muscle stretch by an external force [4], the spindle output can be used to determine the rotation angle of articulation. However, this function cannot be applied when the muscle contracts, because the spindle response to acceleration dominates their response during a passive stretch [5]. When the muscles contract, the Golgi organs respond to the force applied to the tendons, providing information about the muscle activity [6]. Physiological evidence shows that the spindle response is stronger during adaptation tasks, implying that the spindle activity is affected during learning [7,8]. The detailed mechanisms that provide the basal ganglia with the ability to coordinate automatic movements and to adapt were presented in a recent study [9].

Starting from physiological principles and taking into account the increased interest in robotic control using adaptive spiking neural networks (SNNs) [1], in this paper, we present a biologically plausible structure of spiking neurons that is able to control the rotation of a robotic junction and adapt to custom angles of rotation. Note that the goal of this paper is to demonstrate the proposed concept using a reduced number of electronic neurons and not to reproduce the complexity of the biological neural structures in the basal ganglia [9].

The spiking neural network is based on an artificial neuron model of biological inspiration implemented in analogue hardware [10,11]. Electronic circuits represent a better alternative to model the behaviour of biological neurons because this neuromorphic hardware has the main advantages of the natural neurons, such as fully parallel operation and information transmission. Moreover, the variation of internal signals in an infinite range allows the implementation of very complex functions using a reduced number of neurons. Besides these physical similarities between the natural neurons and their electronic models, the latter benefit from very low power consumption and high reliability.

To achieve the smoothness and accuracy of natural motions, artificial muscles should mimic the behaviour of muscular fibres. Thus, in this work, the artificial muscles are implemented with shape memory alloy (SMA) wires that actuate by contraction, as do biological muscles [12,13,14], and their contraction strength can be determined directly by the frequency of the electronic spiking neurons [15,16]. The results reported previously [17] show that, despite the slowness and nonlinearity of SMA wires [18], a small SNN with a bioinspired structure [19] is able to control the rotation angle of a SMA-actuated robotic joint when the arm moves towards target positions. In that case, the spiking neural network behaves as a regulator for the rotation angle, even when the arm is slightly loaded. Moreover, a similar SNN structure can be used as a regulator for the force of SMA actuators when a force sensor (FS) replaces the angle sensor (AS) [20].

## 2. Related Works

Research done until now shows that the contraction of the SMA actuators can be controlled using programmed microcontrollers [21]. Additionally, adaptive SNNs of high biological plausibility were used to control the robotic hands and fingers which were typically actuated by motors [22,23]. The control of SMA actuators using SNNs with fixed weights (non-adaptive) was approached for the first time by our research group [15,16]. As a continuation of this research, the current work presents a new and improved adaptive SNN of high biological plausibility that uses Hebbian learning mechanisms to adapt to custom rotation angles of the robotic junctions.

### 2.1. SMA Actuators

Actuators made of shape memory alloy are suitable for the actuation of anthropomorphic robotic hands [22,24] and other bioinspired systems [14] such as an artificial jellyfish [25], artificial fingers [26], insect legs [27], and wings [28]. Also, various small scale robots are built with Smart Composite Microstructures (SCM) actuated by SMA actuators [29].

### 2.2. Adaptive SNNs

Recently, spiking neural networks have gained a special interest due their performance, reduced signal to noise ratio, and lower power consumption comparative to artificial neural networks (ANNs) [30]. Among the characteristics of the SNN, significant attention is given to their complex adaptability mechanisms [31,32,33,34], which rigorously model the plasticity rules of the biological synapses such as spike timing-dependent plasticity (STDP) [35,36,37,38], input timing-dependent plasticity (ITDP) [39] and homeostasis [40]. Considering that in most practical applications, ANNs show very good performance, several works focus on the conversion of ANNs to SNNs [30]. Also, high performance deep SNNs were implemented with several learning methods [41,42,43] including gradient descent [44,45]. Other learning methods were developed for the detection of spatio-temporal patterns [46,47] and for evolving SNN [48].

### 2.3. SNNs in Robotics

Artificial neural networks were used to describe self-organizing neural models for hand-eye coordination using endogenously generated movement commands correlated with visual, spatial, and motor information to learn internal coordinate transformation [49]. Correlation-based navigation algorithms using STDP learning mechanisms for unsupervised learning were also used to increase the behavioural capabilities of bio-inspired hybrid robots [50]. Robotic arm capability up to 4 degrees of freedom was obtained using an initial period of motor babbling using a spiking neural network architecture that learned autonomously and was simulated according to Izhikevich’s model to exhibit biologically realistic behaviour [23].

In order to apply robot manipulators to a wide class of tasks, it is necessary to control the force exerted by the end-effector on the object along the position of the end-effector. The control of the robot manipulators in the task space was designed with an adaptive neural network based on the inverse dynamic model [51]. The issue of ANN performance in solving inverse kinematics was also approached by the inclusion of the feedback of the current joint angle configuration of robotic arm as well as the desired position and orientation in the input pattern of neural network [52]. There are also studies for determining the Jacobian matrix without knowledge of the forward kinematics of a robotic arm, as well as modifying the Jacobian transpose method to achieve better control stability [53]. Another workaround to avoid the complexity of calculating inverse kinematics and doing motion planning is to use a combination of motor primitives where a SNN may be used to represent motions in a hierarchy of such primitives. Correction primitives may be combined using an error signal to control a robot arm in a closed-loop scenario [54]. To achieve guaranteed tracking control and estimation, an adaptive neural control based on a radial basis function neural network (RBFNN) was also proposed for neural network (NN) weight convergence [55]. 

### 2.4. Adaptive SNN for Motion Control

A reinforced learning mechanism process was used on an artificial motor cortex based on spiking neurons [56]. Using an output that was partially driven by Poisson motor babbling, analogous to the biological dopamine system, a global reward or punishment signal was provided in response to decreasing or increasing the distance from the hand to the target. Dopamine-modulated STDP was also used in an insular cortex model able to detect tactile patterns [57,58,59]. Reinforcement learning for a target reaching task, which can be modelled as partially observable Markov decision processes, may extend the proximal policy optimization using a liquid state machine (LSM) for state representation to achieve better performance [60]. Another example of a learning mechanism based on long-term synaptic plasticity was implemented using the temporal difference learning rule to enable the robot to learn to associate the correct movement with the appropriate input conditions [61]. Another method was also approached, which consists of training a neuromorphic controller online modelled by a leaky integrate-and-fire (LIF) SNN to follow a linear quadratic regulator (LQR) controller with known performance guarantees [62]. Similar work has been accomplished by designing a hierarchical SNN with a bio-inspired architecture for representing different grasp motions. Both the hand and the finger networks were trained independently using STDP, incorporating a mechanism for tactile feedback in the finger networks to stop the motion on contact. For the encoding, values were converted into spikes using a mixture of Gaussian kernels to tune the firing rate of a population. 

Conventional electrical motors were used in [22] for implementing a robotic hand. Another recent paper presents a biomimetic 2-degrees of freedom (DOF) SMA-actuated robotic arm which uses a proportional-integral-derivative (PID) controller to enable closed-loop control of the joint angular positions to prove the technology’s performance against existing commercial DC motor rotary joints [21].

### 2.5. Proposed Concept

The novelty presented in this paper is the structure of an adaptive electronic SNN that is able to learn to rotate the index finger towards the angle intervals where its rotation was blocked previously by an external force. To achieve this goal, the SNN encodes the angle intervals by balancing the excitatory and inhibitory activity and potentiates, using Hebbian learning, the neural paths that correspond to the angle at which the finger was stopped. The proposed adaptive SNN is suitable for being implemented in anthropomorphic robots that are able to learn motions unsupervised in a highly biologically plausible manner. The validation of the proposed concept was performed by physical implementation of a robotic hand with an active index finger. The finger is controlled by the SNN using the feedback from the neuromorphic sensors that convert the joint rotation angle and the compression force into spiking frequency.

The rest of the paper is organized as follows: Section 3 presents the general structure of the bioinspired system focusing on the proposed concept of the adaptive SNN that is validated by simulation in Section 4. The testbed for the evaluation of the proposed SNN architecture is presented in Section 5, which also includes the experimental results and the discussion. The paper ends with Section 6, which discusses the utility of this concept and future research directions.

## 3. Bioinspired System Design

**Figure 1:**
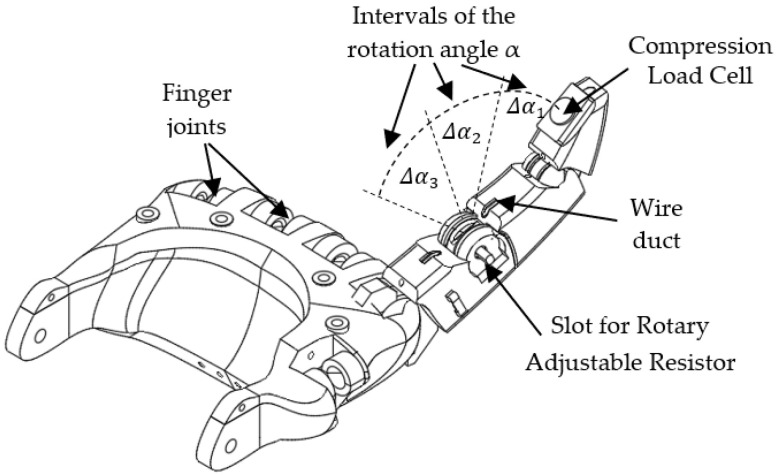
The structure of the anthropomorphic finger.

In order to investigate the performance of the adaptive spiking neural network in controlling the rotation of the robotic junctions, we implemented the robotic hand with active index finger presented in Figure 1.

### 3.1. Artificial Finger

The metacarpophalangeal joint of the robotic finger can be flexed using a SMA actuator. The rotation angle of this joint is converted into voltage by a rotary adjustable resistor (RAR), while the pressure on the finger apex is sensed by a compression load cell (CLC) as in Figure 1.

To protect the finger from heating, the SMA wire is connected near the wrist to a thread that goes towards the apex of the finger. The finger can be blocked by an external force anywhere in the rotation range that includes the angle intervals $\Delta \alpha_{1},\;\Delta \alpha_{2},$ and $\Delta \alpha_{3}$. These angle intervals are delimited by the SNN because it activates one neural path when the finger is in each interval, as we will detail below.

### 3.2. The Structure of the Adaptive SNN

**Figure 2:**
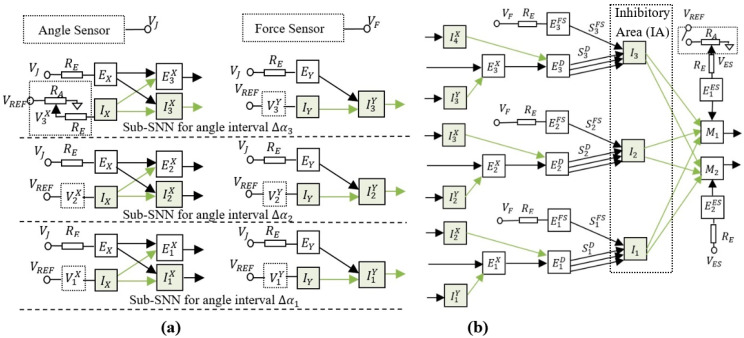
The adaptive neural structure which can be trained using associative learning mechanisms to stop the finger in three subintervals of the rotation range; (**a**) the encoding layers include input neurons $E_{X}$, $E_{Y}$ (excitatory) and $I_{X}$, $I_{Y}$ (inhibitory) that activate a subset of outputs for each rotation interval $∆\alpha_{1-3}$; (**b**) the decoding layers used to activate one of the excitatory neurons $E_{1-3}^{D}$ and adjust the weights of the synapses $S_{1-3}^{D}$ accordingly; the switch determines the activation of the input neurons $E_{1,2}^{ES}$ and, consequently, of the motor neurons $M_{1,2}$.

The spiking neural network presented in Figure 2 is able to learn the intervals where the finger is blocked by an obstacle. To achieve this goal, the neural structure was designed to activate one inhibitory neuron from the inhibitory area (IA) in each of the three sub-SNNs corresponding to angle intervals $\Delta \alpha_{1},\;\Delta \alpha_{2}$ and $\Delta \alpha_{3}$ (see Figure 2a). When the finger reaches the obstacle, the force sensor activates the excitatory neurons $E_{1}^{FS}$, $E_{2}^{FS}$ and $E_{3}^{FS}$ that reduce the activity of motor neurons $M_{1}$ and $M_{2}$ through the inhibitory neurons $I_{1}$, $I_{2},$ and $I_{3}$, respectively. 

Only one of the neurons $E_{1}^{D}$, $E_{2}^{D}$, or $E_{3}^{D}$ is active at the same time, depending on the angle interval in which the finger is rotated. The role of these excitatory neurons is to activate one of the inhibitory neurons that stops the finger in the corresponding angle interval. Note that, if more excitatory neurons would be activated, the finger would stop at the first angle interval it reaches. According to the principles of Hebbian learning, the concurrent activation of more synapses determines their potentiation when the postsynaptic neuron is activated. In this case, the concurrent activation of the potentiated synapses $S_{i}^{FS},\;i=\bar{1,3}$ (activated by FS) with the corresponding un-potentiated synapses $S_{i}^{D}$ (activated when the arm is in the angle interval $\Delta \alpha_{i}$) determines the potentiation of $S_{i}^{D}$ because the neuron $E_{i}^{FS}$ activates the postsynaptic neuron $I_{i}$. Note that, before training, all synapses except $S_{i}^{D}$ have the maximum weights, implying that they determine the activation of the stimulated postsynaptic neurons.

An important structural characteristic of the SNN is related to how the neurons perform sub-interval encoding and decoding. Figure 2a shows the encoding layer that includes excitatory and inhibitory neurons for which the frequency increases with the joint angle α. By analysing the resultant effect of these neurons on the postsynaptic neurons $E_{i}^{D}$, we observed that $E_{i}^{D}$ are activated only when α is in the corresponding angle interval $\Delta \alpha_{i}$. The frequency of the inhibitory neurons $I_{X}$ is set by adjusting the resistors $R_{A}$ in order to ensure the activation of $E_{1}^{X}$ before $E_{2}^{X}$, and $E_{2}^{X}$ before $E_{3}^{X}$ when $V_{J}$ increases. Similarly, a threshold value at which the inhibitory neurons $I_{i}^{Y}$ start to activate is set in order to obtain the activation of $I_{i}^{Y}$ between $E_{i}^{X}$ and $E_{i+1}^{X}$. Also, taking into account that $E_{i}^{D}$ is inhibited when $E_{i+1}^{D}$ is activated implies that voltages $V_{i}^{X}$ and $V_{i}^{Y}$ set the lower and the upper limits of the angle interval $\Delta \alpha_{i}$ where $E_{i}^{D}$ fires, respectively.

### 3.3. Auxiliary Electronics

**Figure 3:**
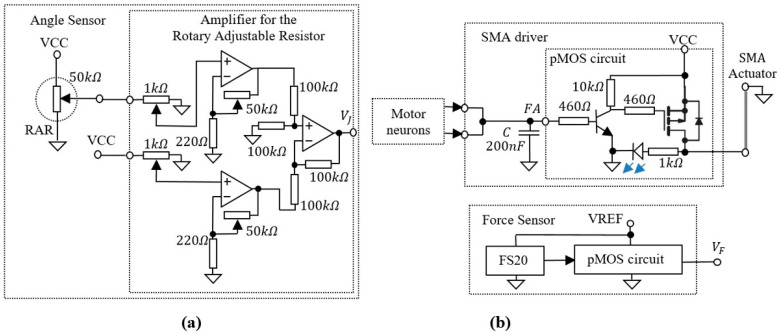
(**a**) Angle sensor including the rotary adjustable resistor and an amplifier; (**b**) SMA driver that integrates the spiking activity of the motor neurons and drives the SMA actuators; A similar pMOS circuit is used to amplify the output of a low force compression load cell FS20.

The bioinspired system includes several auxiliary electronics that perform the adaptation of the analogue signals generated by the sensors to the input or output of the SNN. The electronics presented in Figure 3 include the angle sensor that generates voltage $V_{J}$ for the SNN input, as well as the SMA driver that is used to generate the power for the SMA actuators. 

A similar p-channel MOSFET (pMOS) circuit which is included in the SMA driver is used for output amplification of the compression load cell type FS20.

## 4. Evaluation by Simulation of the SNN Activity

**Figure A1:**
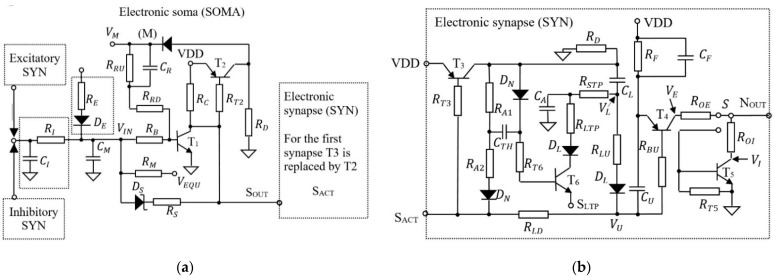
The schematic of: (**a**) the electronic SOMA; (**b**) the electronic synapse (SYN) [11].

The main characteristics of the proposed SNN structure (Figure 2) are the ability to encode angles of rotation by the activation of predefined neural paths and the capability to adapt using Hebbian learning mechanisms. This implies that the synaptic weights are potentiated when the untrained neural paths are activated simultaneously with the trained ones. Prior to hardware implementation of the system, we evaluated by simulation in LT Spice the SNN ability to discriminate the voltage intervals and to adapt by Hebbian learning mechanisms. Using the electronic schematic of the hardware neuron (see Figure A1 in the Appendix A), we simulated the neural network presented in Figure 2b to qualify its behaviour. Since the purpose was only to verify the network in a synthetic environment, all the input infrastructure was replaced by signals generated by voltage sources to mimic real scenarios. The voltage $V_{J}$ was linearly swept through a greater voltage interval that includeed the activation intervals of each excitatory neuron $E_{i}^{D}$. The force sensor output $V_{F}$ was triggered for one value of $V_{J}$ in order to activate the learning mechanism. The activation of $V_{F}$ modeled the presence of an obstacle that pushed on the force sensor in an angle interval. Voltage selectivity was simulated using the potentials $V^{X}$ and $V^{Y}$ that represent the output of simulated voltage generators.

**Figure 4:**
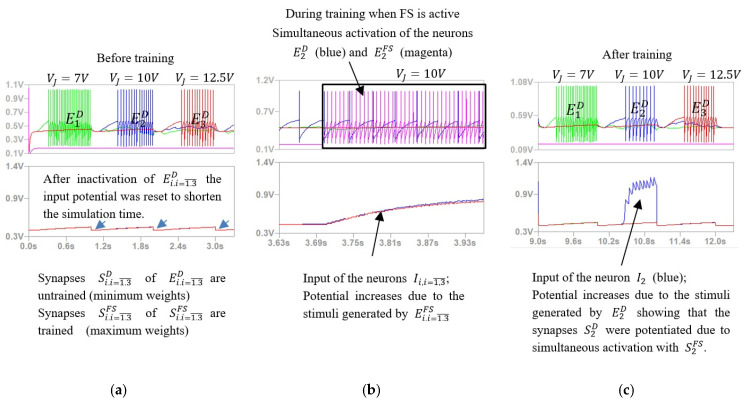
Simulation results showing: (**a**) the activity of the excitatory neurons $E_{1}^{D}$, $E_{2}^{D}$ and $E_{3}^{D}$ (green, blue, and red signals) that respond to predefined voltage levels corresponding to the angle intervals; (**b**) a magenta signal showing the activity of the excitatory neurons that are stimulated by the force sensor (**c**) neuron activity after training, showing the effect of the potentiated synapses that were activated simultaneously with the force sensor.

Figure 4 shows the results obtained during the simulation of SNN activity when the force sensor was activated concurrently with neuron $E_{2}^{D},$ whose activity simulates the the finger positioning in the angle interval $\Delta \alpha_{2}$. The upper signals represent the activity of the excitatory neurons $E_{1}^{D}$, $E_{2}^{D}$ and $E_{3}^{D}$ (Figure 2b) that stimulate the corresponding postsynaptic inhibitory neurons $I_{1}$, $I_{2}$ and $I_{3}$ for which the input is shown by the lower signals. 

Note that, the activation of the neurons $E_{i}^{D}$ depends on the value of $V_{J},$ implying that the SNN can discriminate between angle intervals (Figure 4a,c). Moreover, as presented in Figure 4c, after training, the stimulation of the neuron $I_{2}$ by the neuron $E_{2}^{D}$ was significantly stronger than the stimulation of $I_{1}$ and $I_{3}$ by $E_{1}^{D}$ and $E_{3}^{D}$, respectively. This shows that the concurrent activation of neurons $E_{2}^{D}$ and $E_{2}^{FS}$ (Figure 4b) potentiates only the synapses $S_{2}^{D}$, while the weights of $S_{1}^{D}$ and $S_{3}^{D}$ remained low. 

## 5. Experimental Investigation

The simulation results illustrate that the SNN behaved as expected, allowing us to test these abilities in hardware, as well as the performance of the SNN in stopping the finger in the corresponding angle interval after training.

### 5.1. Experimental Setup

**Figure 5:**
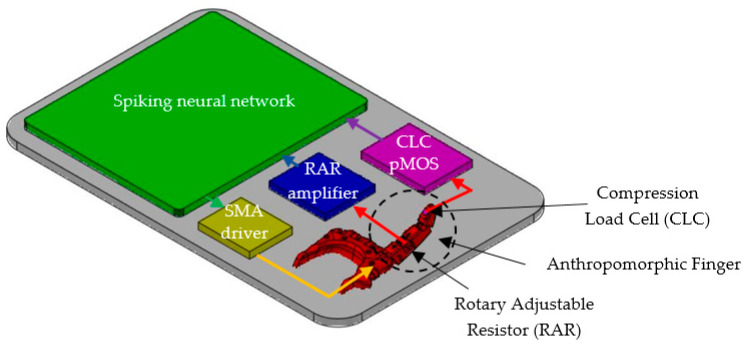
Experimental setup showing the structure of the bioinspired system which includes the spiking neural network, the artificial finger, and auxiliary electronics (SMA driver, RAR amplifier and the CLC pMOS).

Figure 5 shows the structure of the bioinspired system including the adaptive SNN that was able to detect the presence of the potential $V_{J}$ (generated by the angle sensor) in three different voltage intervals and to learn which neural path fired when the force sensor was active. The SNN controlled the actuators through the SMA driver and received information about the rotation of the artificial finger from the RAR amplifier and about the applied force on the finger apex from the CLC pMOS (Figure 3b).

The artificial finger was flexed by an 82 cm-long SMA Flexinol 0.006”-type actuator for which the maximum load was 321 g at 410 mA and cooling time was 2 s. The reference voltages for the SNN are $V_{EQU}=0.4\;V$ and $V_{REF}=5\;V$, the later potential being used also to power the force sensor. The supply voltage for the neurons was VDD=1.6 V, while the SMA actuators and angle sensor were powered by VCC=14 V. During the experiments, the room temperature was about 23 °C.

### 5.2. Experiments Overview

The main characteristics of the SNN that were tested in the next were angle interval selectivity and SNN adaptability by associative learning mechanisms. For the selectivity evaluation we monitored the activity of neurons $E_{1}^{X},$ $I_{1}^{Y}$, and $I_{2}^{X},$ included in the sub-SNN which was used to detect the finger in the angle interval $∆\alpha_{1}$. When $V_{J}$ was in the potential variation range for $∆\alpha_{1}$ the sub-SNN output was activated. Also, we monitored the activity of neurons $E_{2}^{D}$ and $E_{3}^{D}$ when $V_{J}$ took several values in the corresponding intervals $∆\alpha_{2}$, $∆\alpha_{3}$ and, respectively, the transition between them. 

SNN adaptability was evaluated by monitoring $V_{J}$ and the activity of neurons $E^{D}$, I, and $E^{FS}$ when the finger was actuated. Before training, no inhibition occurred, and during training, the neuron activity showed that the finger pushed on the obstacle rhythmically. After training, $E^{D}$ activates the postsynaptic neurons I in the absence of the obstacle, stopping the finger. Also, we showed that the inhibitory neurons $I_{1}$, $I_{2},$ and $I_{3}$ were able to independently stop the finger rotation at different angles of rotation.

### 5.3. Experimental Results

**Figure 6:**
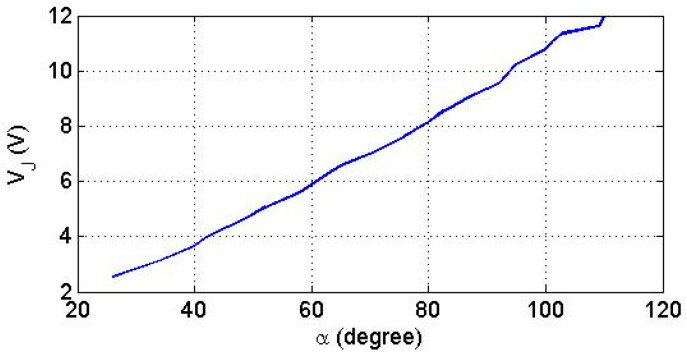
The function $V_{J}$(α) generated by the angle sensor.

Considering that angle α directly determines the voltage $V_{J}$ generated by the angle sensor, we evaluated the function $V_{J}\left(\alpha \right)$ experimentally which is plotted in Figure 6. 

Considering that the linearity of the function $V_{J}\left(\alpha \right)$ is high, we will refer below only to the voltage $V_{J}$ in order to simplify the presentation of the results. Thus, the angle intervals $\Delta \alpha_{1-3}$, correspond to the voltage intervals $\Delta V_{1-3}$ of the voltage $V_{J}$.

#### 5.3.1. Voltage Interval Selectivity

**Figure 7:**
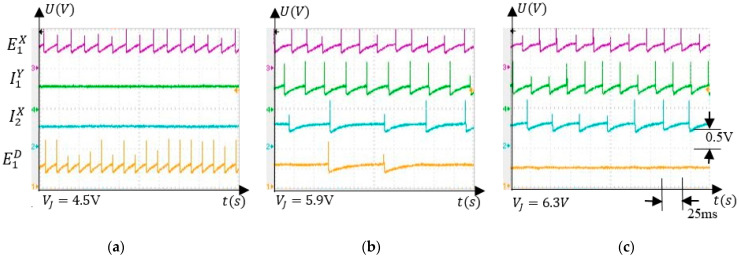
Activity of the neurons that stimulate $E_{1}^{D}$ when $V_{J}$ takes several values; (**a**) no inhibition; (**b**) partial inhibition; (**c**) ful inhibition.

To evaluate the response of the neurons to $V_{J},$ the variation of the finger was positioned by an external force in $\Delta V_{1}$. Figure 7 presents the electronic neuron activity for several values of $V_{J}$ which were chosen to highlight the SNN ability to detect that $V_{J}$ was in $∆V_{1}$. Note that the spikes on the diagrams represent electronic neuron activations and the signals represent the potential $V_{M}$ recorded using a TDS2024 oscilloscope in node (M) of the neuron’s schematic (see Appendix A). In Figure 7a, the excitatory neuron $E_{1}^{X}$ determines the activation of $E_{1}^{D}$ in the absence of inhibition produced by the neurons $I_{1}^{Y}$ and $I_{2}^{X}$. As the inhibitory activity becomes stronger, the frequency of $E_{1}^{D}$ reduces (Figure 7b) until it is fully inhibited, as in Figure 7c. 

**Figure 8:**
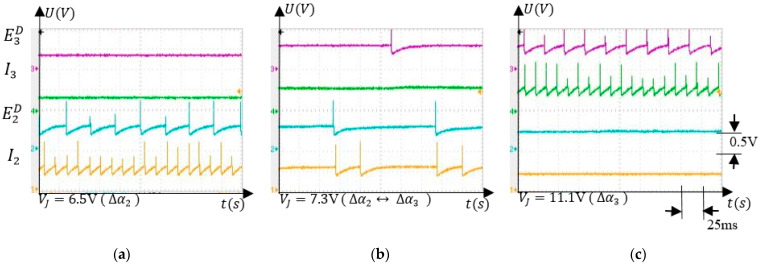
Neuron activity that shows the ability of the SNN to select which neural path to activate according to $V_{J}$: (**a**) $E_{2}^{D}$ and $I_{2}$ activated when the finger is in $∆\alpha_{2}$; (**b**) neurons from both neural paths are activated when the finger is between $∆\alpha_{2}$ and $∆\alpha_{3}$; (**c**) $E_{3}^{D}$ and $I_{3}$ are activated when the finger is in $∆\alpha_{3}$.

The ability of the SNN to activate neural paths that are specific to the values of the input voltage is highlighted by the signals shown in Figure 8. The full activation of neurons, $E_{2}^{D}$ and $E_{3}^{D}$ occurs when $V_{J}=6.5\;V$ (Figure 8a) and $V_{J}=11.1\;V$ (Figure 8c), respectively.

Taking into account that $V_{J}$ is generated by the angle sensor, the activation of $E_{2}^{D}$ and $E_{3}^{D}$ signal the presence of the finger in the angle intervals $\Delta \alpha_{2}$ and $\Delta \alpha_{3}$, respectively. When signal $V_{J}$ crosses between thevintervals $∆V_{2}$ and $∆V_{3,}$ both $E_{2}^{D}$ and $E_{3}^{D}$ are activated at a lower frequency. 

#### 5.3.2. Associative Learning

**Figure 9:**
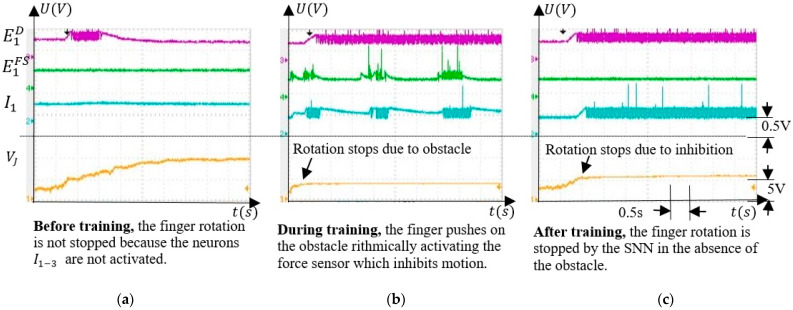
(**a**) Before training, neuron $E_{1}^{D}$ detects the presence of the finger in the corresponding angle interval; (**b**) during training, neuron $E_{1}^{D}$ is activated simultaneously with the neuron $E_{1}^{FS}$ activating the neuron $I_{1}$; (**c**) after training, neuron $E_{1}^{D}$ activates inhibitory neuron $I_{1}$ stopping the finger rotation without the activity of the $E_{1}^{FS}$ neurons.

The main feature of the SNN is the ability to adapt to activate the corresponding neuron $I_{i},\;i=\bar{1,3}$, which inhibits motor neurons $M_{1,2},$ stopping the finger’s rotation (Figure 2b). Learning occurs by long term potentiation when the unpotentiated synapses $S_{i}^{D}$ are activated simultaneously with the potentiated synapses $S_{i}^{FS}$ that activate $I_{i}$. Therefore, the SNN training consisted of potentiating the excitatory synapses $S_{i}^{D}$ that connect the neurons $E_{i}^{D}$ to the inhibitory neurons $I_{i}^{D}$. The weights of $S_{i}^{D}$ increase when the force sensor is activated if the finger is in the corresponding angle interval. An example of the neuron activity when the force sensor is activated by an obstacle in the interval $∆\alpha_{1}$ is shown in Figure 9. Before the training, neuron $E_{1}^{D}$ detected that the finger crosses the corresponding angle interval without stopping it (Figure 9a). As shown in Figure 9b, during training, the neuron $I_{1}$ is activated only by $E_{1}^{FS}$ and not by $E_{1}^{D}$, which fires continuously because the finger is stopped in $∆\alpha_{1}$. The activity of neuron $E_{1}^{FS}$ alternates with silent periods, showing that the SNN has a regulatory behaviour consisting of trying to push on the obstacle. 

After training, the force sensor remained inactive because the obstacle was removed implying that the neuron $E_{1}^{FS}$ was silent, as presented in Figure 9c. The neuron $I_{1}$ was activated only by the neuron $E_{1}^{D}$ through the potentiated synapses $S_{1}^{D}$. Denoting the potential where the finger was stopped by the external force as $V_{J}^{EF}$, and the value where the finger stops due to inhibition after training as $V_{J}^{INH}$, one can observe that $V_{J}^{EF}\neq V_{J}^{INH}$.

This occurs because the finger can be stopped by an obstacle anywhere in the angle interval, but the SNN will stop the finger where the inhibitory activity of $I_{1}$ compensates for the excitatory output of neurons $E_{1,2}^{ES}$ (Figure 2b).

#### 5.3.3. Finger Operation

**Figure 10:**
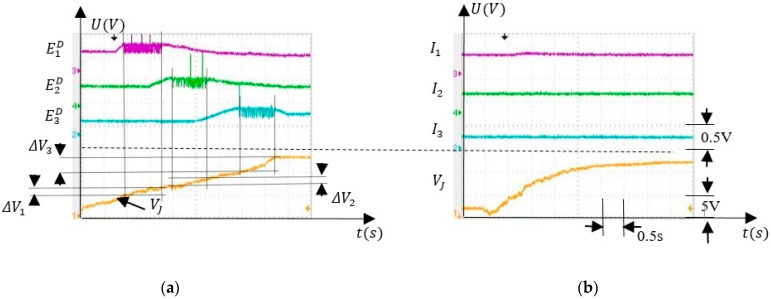
The activity of the critical neurons before training showing the angle interval selection; (**a**) $E_{1-3}^{D}$ when the artificial finger was rotated by the hand; (**b**) $I_{1-3}$ when the SMA actuator rotated the finger.

**Figure 11:**
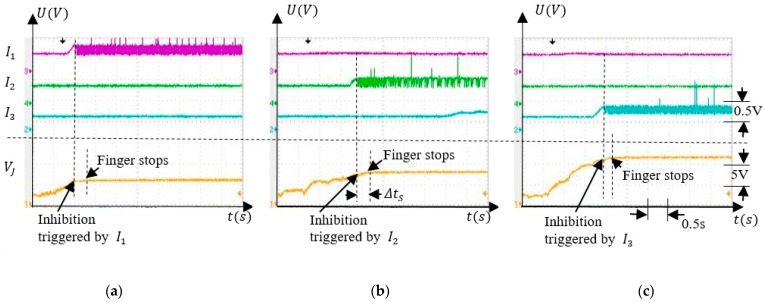
The activity of the inhibitory neurons $I_{1-3}$ stops the finger rotation at angle α corresponding to the values of $V_{J}$: (**a**) $I_{1}$; (**b**) $I_{2}$; and (**c**) $I_{3}$.

To test the behaviour of the SNN when the finger was rotated, we focused on the independent activity of the inhibitory neurons $I_{1-3}$. when only one of these neurons fires. First, we tested if the SNN was able to discriminate the angle intervals $∆\alpha_{1-3}$ when the finger was actuated by an external force, as presented in Figure 10a. Second, the weights were set to their minimum values when the inhibitory neurons were not sensitive to the finger rotation as in Figure 10b. Figure 11 presents the activity of the inhibitory neurons when only the synapses $S_{i}^{D}$ were trained.

Note that the potential $V_{J}$ remains stable shortly after activation of $I_{i},$ implying that the finger stopped in the interval $∆\alpha_{i},$ where the force sensor was activated during training. The values where $V_{J}$ remained constant depended on which inhibitory neuron was activated. Considering that $V_{J}$ was constant when the rotation was stopped, implies that the corresponding angle $\alpha_{i}$ depended on the activated neurons $I_{i}$, as expected. Also, there is was a $∆t_{S}$ delay between the activation of the inhibitory neurons and the moment at which the rotation stopped which was determined by the cooling time of the SMA actuator.

### 5.4. Discussions

The results show that the SNN was able to rigorously discriminate several voltage intervals of the input by balancing the activity of the excitatory and inhibitory neurons despite the oscillations of the finger speed. Also, using few neurons, the SNN learned to activate the inhibitory neurons according to the angle interval where the finger was stopped by the external force. In this work, we did not focus on the precision and accuracy of the finger positioning because these parameters were analysed previously using a similar system based on SNN and SMA actuators [17]. Also, the SNN was not able to stop the finger exactly at the same angle at which it was blocked by the obstacle because the angle intervals considered were wide. The performance of the finger positioning can be increased by narrowing the angle intervals that can be obtained using more neurons. Theoretically, the minimum width of the angle intervals $∆\alpha_{min}$ increases with the rotation speed of the finger joint, implying that lower speeds allow better resolution. As an example, when the rotation speed is 40 °/s, the maximum rotation range of $\alpha_{range}=$ 120° is covered by the finger in 3 s. By increasing the number of inhibitory neurons and by significantly reducing the cooling time of the SMA actuator (using water with glycol), the finger can stop when the inhibitory neurons fire once. The simulations of the SNN activity show that the minimum variation of $V_{J}$ that activates the inhibitory neurons once is $∆V_{min}=0.25$ V, which corresponds to $∆\alpha_{min}=$ 5.8°. This implies that the maximum number of intervals that cover $\alpha_{range}$ is about $n_{max}=20$.

The SNN performance was evaluated when the finger was flexed. Knowing that the inhibitory neurons fire when the finger is positioned in the corresponding angle interval independent of the previous one, we can consider that associative learning and inhibition also occur when the finger is rotated in the opposite direction.

Another observation is related to the position of the obstacle at the edge of the angle intervals. In this setup, the intervals $∆\alpha_{1-3}$ are disjunctive, implying that both neurons $E_{i}^{D}$ and $E_{i+1}^{D}$ fire at a lower frequency when the finger crosses from $∆\alpha_{i}$ to $∆\alpha_{i+1}$ (Figure 8b). This behaviour of the SNN reduces the learning rate, implying that in this uncertainty case, the synapses potentiation is insignificant. However, the intersection of the intervals $∆\alpha_{i}$ and $∆\alpha_{i+1}$ can be obtained by making the activation of the neurons $E_{i}^{X}$ and $I_{i}^{X}$ independent (see Figure 2a) and by setting accordingly the intervals limits. When the obstacle is placed at the intersection of $∆\alpha_{i}$ and $∆\alpha_{i+1}$, both $E_{i}^{D}$ and $E_{i+1}^{D}$ activate, potentiating $S_{i}^{D},$ and $S_{i+1}^{D}$ will stop the arm in the first interval that is reached, reducing the positioning resolution to half.

## 6. Conclusions

The experiments demonstrate that a bioinspired control system based on an adaptive neural structure of biological inspiration and contractile SMA actuators is sensitive to the rotation angle of an anthropomorphic finger. This is achieved by the activation of different neural paths for different values of the input potential that correspond to several angle intervals. When a supraliminar stimulus activates all neurons that inhibit the output, and thus stopping the rotation, the SNN learns to determine which angle detection neural path (ADNP) was active. This adaptation mechanism connects the ADNP to one inhibitory neuron that stops the rotation in the absence of the supraliminar stimulus. Taking into account the high level of bioinspiration given by the spiking neural structures that control the contractile actuators, this concept can be used to understand how the automatic motions are gained in the basal ganglia. Also, anthropomorphic robots that learn motions based on biological principles could benefit from this concept. Another advantage of this system is the implementation of a spiking neural network in analogue hardware that allows for the control of multiple actuators in parallel without affecting the real-time response of the system.

As a short-term goal, we will comparatively evaluate the performance of SNN and microcontrollers in controlling in parallel the SMA actuated junctions of an anthropomorphic hand for showing in which conditions SNN represents a more advantageous control method. Another future direction is to design a neuron model with an improved learning mechanism in terms of biological rigor and to implement it on an FPGA, which simplifies the prototyping of the future SNN structures.

## Data Availability

Not applicable.

## Appendix A

Figure A1 presents the schematic circuit of the electronic neuron that is implemented in PCB hardware [10,11]. The neuron includes one electronic soma (SOMA) and one or more electronic synapses (SYN). The SOMA detects neuron activation threshold using the transistor $T_{1}$ and activates the SYNs. As presented in Figure A1a the SOMA of the input neurons which are connected directly to the analogue signals ($V_{1-3}^{X}$, $V_{1-3}^{Y}$, $V_{F}$, $V_{J}$ and $V_{ES}$ in Figure 3) includes the pair $R_{E}$–$D_{E}$ that determine the oscillation of the neuron with frequency f that depends on the input potential. The SOMA of the postsynaptic neurons which are stimulated by the excitatory or inhibitory synapses includes the pair $C_{I}$–$R_{I}$ which integrates the input activity. When the SOMA activates the connected SYNs, $S_{OUT}$ generates pulses at their output $N_{OUT}$, whose energy depends on the charge stored in the weight capacitor $C_{L}$. Note that the potential $V_{M}$ was monitored using the oscilloscope in node (M) for visualization of the neuron activations, which are represented by the spikes. Depending on the position of switch S, the generated pulses can be excitatory with maximum amplitude VDD, or inhibitory with minimum amplitude GND (see Figure A1b).

The parameters of the electronic neuron (SOMA and SYN) that were used in the experiments are given in the Table A1 and Table A2.

**Table A1:** Parameters for the SOMA.

Param.	Value	Param.	Value
R_RU_	20 kΩ	T_1_	BC848C
R_RD_	1 kΩ	T_2_	BC857C
R_IN_	220 kΩ	D_S_	BAR43
R_B_	6.2 kΩ	D_N_	1N4148
R_C_	10 kΩ	D_L_	BAS45A
R_D_	1 MΩ	C_R_	10 nF
R_M_	1 MΩ	C_I_	1 μF
R_S_	47 Ω	C_M_	100 nF
R_T2_	10k Ω		

**Table A2:** Parameters for the SYN.

Param.	Value	Param.	Value
R_D_	1 MΩ	C_TH_	10 nF
R_F_	47 kΩ	C_A_	47 nF
R_T3_	10 kΩ	C_L_	2.2 μF
R_A1_	10 kΩ	C_U_	221 pF
R_A2_	1 kΩ	C_F_	1 μF
R_T6_	470 Ω	T_3_	BC857C
R_STP_	10 kΩ	T_4_	BC857C
R_LTP_	470 Ω	T_5_	BC848C
R_OE_	1.8 kΩ	D_N_	1N4148
R_LU_	1 MΩ	D_L_	BAS45A
R_LD_	470 kΩ	R_E_	560 kΩ
R_BU_	10 kΩ	R_A_	5 kΩ
R_OI_	470 Ω		
R_T5_	47 kΩ		
